# Patients with Robotic Arm-Assisted Medial Unicompartmental Knee Arthroplasty (mUKA) Regain Their Preoperative Activity Level Two Weeks Earlier Compared to Robotic Arm-Assisted Kinematically Aligned Total Knee Arthroplasty (rKA-TKA)

**DOI:** 10.3390/s25061668

**Published:** 2025-03-08

**Authors:** Carlo Theus-Steinmann, Sietske Witvoet-Braam, Kim Huber, Sarah Calliess, Bernhard Christen, Tilman Calliess

**Affiliations:** 1 Articon Spezialpraxis für Gelenkchirurgie, Berner Prothetikzentrum BPZ, 3013 Bern, Switzerlandt.calliess@articon.ch (T.C.); 2 Stryker, Digital, Robotics, & Enabling Technologies, 1101 CM Amsterdam, The Netherlands

**Keywords:** rehabilitation, Garmin Vivofit 4, step count, robotic-assisted, MAKO, individualized alignment, FJS

## Abstract

Background: This study compared the early rehabilitation progress of patients undergoing robotic-assisted medial unicompartmental knee arthroplasty (mUKA) and robotic-assisted kinematically aligned total knee arthroplasty (rKA-TKA), focusing on daily activity by step-count measurements. Methods: A retrospective analysis of prospectively collected data from 88 patients (53 rKA-TKA and 35 mUKA) was conducted. Patients wore Garmin Vivofit^®^ 4 activity trackers pre and postoperatively. Daily step counts were analyzed, and clinical outcomes were assessed using various scores, including the Knee Society Score (KSS) and Forgotten Joint Score (FJS). Results: Preoperative median daily step counts were comparable between groups (rKA-TKA: 3988 and mUKA: 4315; *p* = 0.128). At 6 and 7 weeks post-surgery, the mUKA group showed significantly higher median step counts (3741 and 4730) compared to the rKA-TKA group (2370 and 2910), with *p*-values of 0.015 and 0.048, respectively. The mUKA group reached 86.7% of their preoperative step count at week 6 and 100% at week 7, while the rKA-TKA group achieved 59.4% and 73%, respectively. Both groups surpassed their preoperative activity levels by week 9. Clinical outcomes at 2 months and 1 year post-surgery showed no significant differences between groups. Conclusions: While both the mUKA and rKA-TKA patients achieved their preoperative daily activity levels within nine weeks post-surgery, the mUKA patients reached this milestone approximately two weeks earlier. This study demonstrates a clinical benefit of mUKA in terms of faster postoperative remobilization, even when compared to kinematically aligned robotic-assisted TKA.

## 1. Introduction

Knee arthroplasty is a well-established and clinically successful treatment for end-stage osteoarthritis, and there is a growing trend worldwide of treating even younger and more active patients with this approach. Patients’ expectations for an early return to activity and ability to perform sports after joint replacement are also increasing. Rapid rehabilitation after surgery generally reduces healthcare costs, lowers the occurrence of complications, and, especially for patients who are still working, has an important economic impact by improving a patient’s capacity to return to work after surgery.

In this context, there is already some evidence that unicompartmental knee arthroplasty (UKA) offers superior results to total knee arthroplasty (TKA) in the postoperative period [1,2,3,4]. Fewer perioperative complications are reported for UKA, especially for infections and cardiovascular events [4]. Additionally, hospital discharge and postoperative rehabilitation are often faster, as noted in numerous studies [4,5]. Furthermore, patients with UKA return to sport earlier and are more likely to forget their artificial joint in daily life compared to those with TKA [6,7,8,9].

In addition to the perioperative management and the type of the prosthesis, surgical management, precision, and invasiveness might also play a role in the rehabilitation process. In this context, haptic robotic arm-assisted technologies have been introduced as one major evolution in knee arthroplasty. Numerous studies have demonstrated a lower complication and revision rate, especially for UKA compared to manually implanted controls [10]. Several studies have also shown that image-based haptic robotic systems reduce soft tissue trauma, resulting in a lower level of postoperative pain. This, in turn, can be a key factor in promoting rapid postoperative rehabilitation. Lastly, robotic-assisted technology has been shown to enable the precise implementation of new, personalized alignment philosophies in knee arthroplasty, with lesser soft tissue releases and a more physiological joint reconstruction [11,12]. Once again, this is an important factor for faster rehabilitation, as stated in numerous studies.

One unanswered question is whether individualized, robotic arm-assisted total knee arthroplasty can enable a rehabilitation duration similar to that of a robotic arm-assisted unicompartmental knee arthroplasty. However, to answer this question meaningfully, one must think about an appropriate methodology.

Currently, there is little evidence on the time it takes for patients to return to their individual preoperative activity level after any kind of knee arthroplasty. Classic outcome measurement usually focuses on patient-reported outcome measures (PROMs), standardized questionnaires given to the patient at predefined time points, often 3, 6, and/or 12 months postoperative. These PROM scores might miss subtle differences in the initial mobilization phase postoperatively. In this context, an unsupervised real-world observation of patient activity, as can be achieved with modern smartwatches or pedometers, appears much more interesting and meaningful. Activity trackers provide objective data on physical activity levels, gait parameters, and functional outcomes, complementing traditional PROMs. Studies have shown that wearable sensors can track improvements in daily steps, range of motion, and load asymmetry during the postoperative period. This technology allows for remote monitoring of patients, potentially enhancing rehabilitation protocols and providing more comprehensive insights into recovery patterns after both total and partial knee arthroplasty procedures [13,14,15]. While faster recovery with UKA might be expected due to its less invasive nature, we hoped to offer substantial added value with this study by quantifying this difference precisely within the context of modern robotic-assisted techniques for both procedures.

The aim of this study was to compare the early rehabilitation progress in patients undergoing robotic arm-assisted unicompartmental and total knee arthroplasty, with a focus on their daily activity, quantified by step-count measurements. By comparing these two robotic-assisted techniques, we aimed to evaluate their relative efficacy in facilitating patients’ return to preoperative activity levels, potentially informing clinical decision making and improving patient outcomes in knee arthroplasty procedures. We hypothesized, based on our clinical observations, and in the absence of existing literature, that patients receiving robotic arm-assisted medial unicompartmental arthroplasty (mUKA) regain preoperative daily activity levels faster than those undergoing robotic arm-assisted kinematically aligned total knee arthroplasty (rKA-TKA).

## 2. Materials and Methods

This study retrospectively reviewed the prospectively collected data of patients undergoing robotic arm-assisted medial unicompartmental or robotic arm-assisted kinematically aligned total knee arthroplasty in our department between 07/2019 and 06/2021 (Mako, Stryker, Fort Lauderdale, FL, USA). Inclusion criteria were informed consent for data collection, analysis, and publication, a full record of patients’ demographics and routine outcome measurements in our institute’s database, the indication for surgery due to symptomatic primary osteoarthritis (OA), plus the voluntary participation in our program to wear an activity tracker in the perioperative period. Exclusion criteria were any kind of secondary or post-traumatic OA, and a preoperative activity level lower than “regularly participating in leisure activities such as swimming or gardening”, as well as any other type of partial knee implant (lateral unicompartmental, patellofemoral, or bi-unicompartmental knee arthroplasty). The indication for UKA or TKA was independent from the study, based on the wear pattern and the institution’s standard indication criteria. In our department, unicompartmental knee replacements constitute 40% of all knee replacement procedures, with medial unicompartmental knee replacements specifically accounting for 30% of all knee arthroplasty procedures. The philosophy for UKA was primary a one-to-one surface reconstruction and balancing with the robotic navigation, as described before [16]. The TKA followed the concept of restricted kinematic alignment (rKA), as described before [17].

All of the patients were assessed preoperatively in the outpatient clinic with the Knee Society Score (KSS), Knee Injury and Osteoarthritis Outcome Score (KOOS), Oxford Knee Score (OKS), and the EuroQol 5-Dimension 5-Level (EQ-5D-5L). At 2 months and 1 year postoperatively, there was a visit in the outpatient clinic, again with the assessment of before-mentioned instruments, plus the Forgotten Knee Joint Score (FJS).

At their preoperative assessment, patients were given a Garmin Vivofit^®^ 4 (Garmin, Olathe, KS, USA) activity tracker to wear until the day of surgery to assess their preoperative daily step count. The device is designed to be worn on the wrist, has a long battery life, which is an advantage for continuous monitoring, and has a high accuracy for recording step counts [18,19]. Preoperative data were extracted from the device on the day of surgery.

After discharge from hospital (3–5 days postoperatively), the patients continued to wear the device. Postoperative data were collected and then extracted for analysis at the first postoperative assessment (scheduled 6–10 weeks after surgery). Due to data storage limitations, the device would overwrite previous measurements with new measurements when the capacity limits were reached. A maximum of 42 measurements (days) could be stored.

The daily pre- and postoperative step count data were extracted from the Garmin device. The daily measurements were converted into an average weekly step count by taking the median value of all available measurements within consecutive weeks post-surgery.

In the analysis of preoperative and postoperative step-count measures between the two groups, we employed different statistical methods based on data distribution. For continuous variables with normal distributions, we utilized Student’s t-test for comparisons. In contrast, the Mann–Whitney U test was applied to compare continuous variables that did not follow a normal distribution. We opted not to perform significance tests for intervals where either group had very few measurements (less than 10).

For presentation purposes, we reported normally distributed continuous variables using the mean and standard deviation. For variables that did not exhibit a normal distribution, we presented the median and interquartile range (IQR). Categorical variables were represented as the percentage of patients experiencing the outcome. We established statistical significance at a *p*-value below 0.05 for all of the analyses. All statistical computations and analyses were conducted using Python version 3.10, using the statistical module of the scientific Python library scipy.stats [20].

## 3. Results

**Figure 1:**
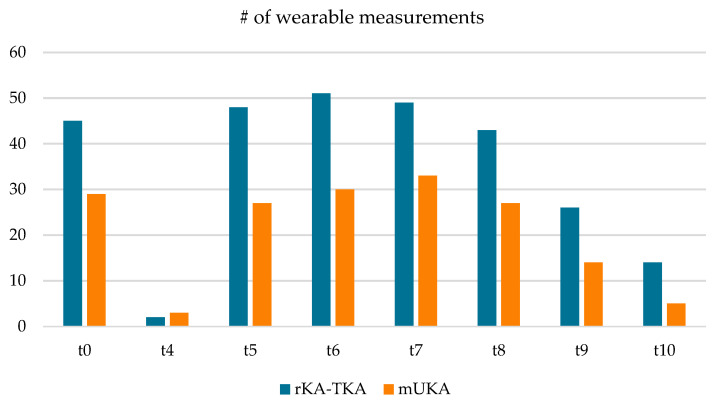
Number of wearable measurements is shown for each time interval (t0: preoperative; t4–t10: postoperative week 4–week 10) for rKA-TKA and mUKA. Due to the storage capacity of the device, the number of assessments per weekly interval varied strongly.

Out of the 88 included patients, activity tracker measurements were successfully recorded for 84 patients. Among these, 49 patients received a total knee arthroplasty (TKA) and 35 patients received a medial unicompartmental knee arthroplasty (mUKA). Preoperative and postoperative data were available for 74 patients (45 TKA and 29 UKA). On average, activity was recorded for 21 days preoperatively (range 8–42 days) and 28.7 days postoperatively (range 17–42 days) between weeks 4 and 10 following surgery (Figure 1).

### 3.1. Demographics

There was no statistical difference in patient demographics between patients receiving a total knee arthroplasty and those receiving a medial partial knee arthroplasty, except for the ASA morbidity state (Table 1).

Patients in the total knee group were slightly older and a larger proportion was female, without statistical significance. There was no difference in BMI between both groups. A total of 51% of the partial knee group were in the two highest physical demand levels (C and D) compared to only 32% of the total knee group.

### 3.2. Activity Data

**Figure 2:**
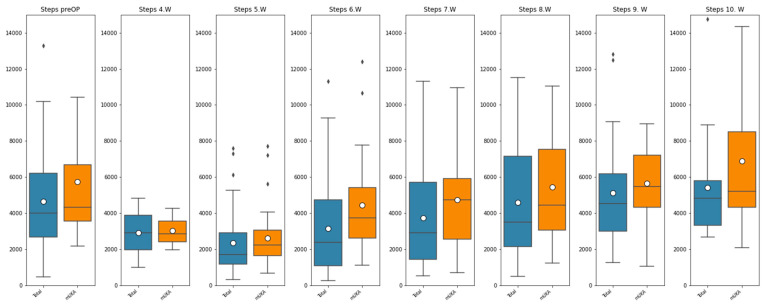
Boxplot of median step count at each interval. ♦ Outliers. Blue: rKA-TKA and orange: mUKA.

Step-count data for both groups were not normally distributed at any time interval. The range and median step count for both groups are presented in Table 2 and Figure 2.

Prior to surgery, the median step count per day for both groups was comparable (TKA: 3988 vs. mUKA: 4315; *p* = 0.128). In the early postoperative weeks, both groups showed a decline in the average daily step count. At postoperative weeks 6 and 7, the data showed a significant difference in the median step count per day in favor of the mUKA group (3741 and 4730 versus 2370 and 2910; *p*-values of 0.015 and 0.048).

Each boxplot in Figure 2 visually represents the corresponding study outcome. The horizontal line within the box indicates the median, while the white dot represents the mean. The box itself illustrates the interquartile range (IQR). The whiskers extend to the minimum and maximum values, except for data points that fall more than 1.5 times the IQR below the lower quartile or above the upper quartile; these outliers are displayed separately.

**Figure 3:**
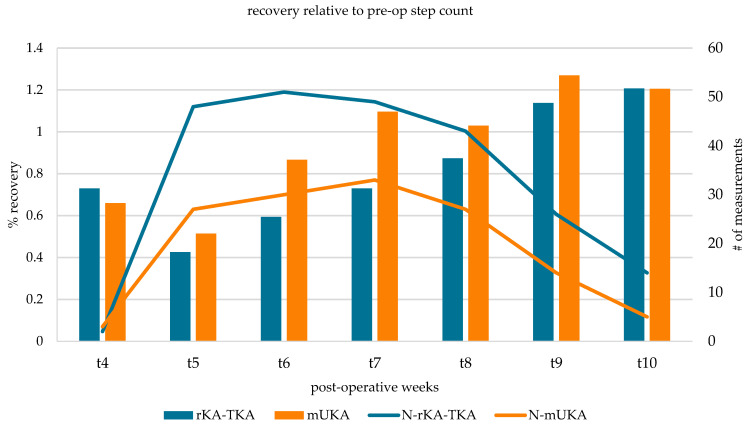
Percentage of the step count recovery per group and interval. Lines represent number of measurements.

Figure 3 shows the postoperative recovery toward the preoperative median step count for both groups, along with the number of measurements at each interval. At week 6, the medial unicompartmental knee group reached 86.7% of the group’s median preoperative step count, while the total knee group was at 59.4% of their preoperative state. At 7 weeks, the medial unicompartmental knee group reached their preoperative state, while the total knee group was at 73% of their preoperative state. At these two intervals, the difference in the step count was significantly different between both groups. At week 9 and beyond, both groups were more active than before surgery.

### 3.3. Clinical Outcomes

**Figure 4:**
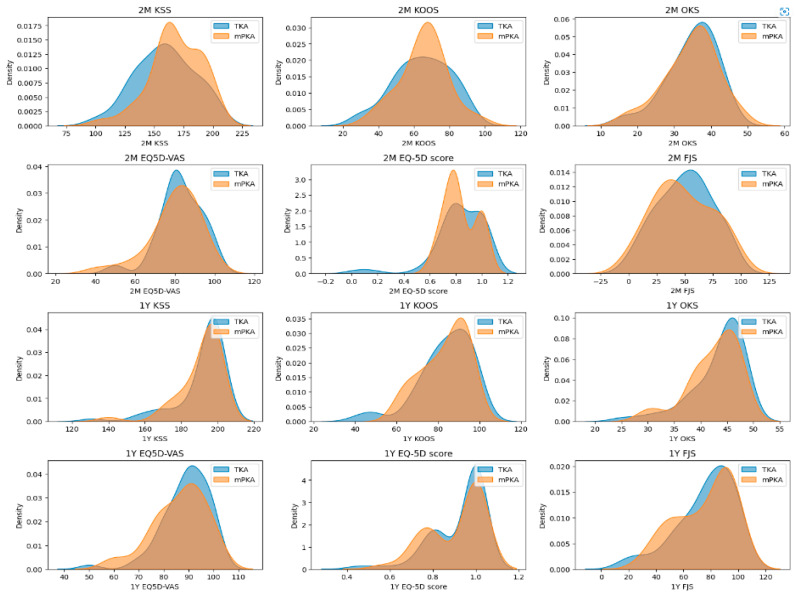
Kernel density plots of PROMS at 2 months and 1 year per group.

At the 2-month and 1-year follow-ups, there were no differences in clinical outcomes regarding the KSS, KOOS, OKS, EQ-5D-5L, or FSJ between the two groups, as displayed in Table 3 and Figure 4.

## 4. Discussion

The aim of this study was to compare the rehabilitation rate in patients undergoing robotic-assisted unicompartmental or total knee arthroplasty by quantifying the daily activity through step-count measurements. The most important finding was that, although the clinical outcome at 2 month and 1 year postoperative did not differ between the two groups in the established outcome scores, the mUKA patients showed a significantly faster postoperative remobilization, achieving their preoperative activity level 2 weeks faster than the TKA patients. Thus, we were able to prove our hypothesis and demonstrate a clinical benefit of UKA, even when compared to robotic arm-assisted kinematically aligned TKA, which has been demonstrated to significantly improve outcomes and rehabilitation in TKA [21].

The clinical outcomes of robotic arm-assisted unicompartmental knee arthroplasty (RA-UKA) and robotic arm-assisted kinematic alignment total knee arthroplasty (RA-KA TKA) remain limited in the literature. Among the available patient-reported outcome measures, the Forgotten Joint Score (FJS) stands out as the most frequently published and, due to its low ceiling effect, the most valuable clinical outcome.

Our RA-UKA outcomes revealed (median) FJS scores surpassing those reported by Blyth et al. by 10 to 15 points at both the 2- and 5-year postoperative intervals [22,23]. This notable disparity may be attributed to the patient cohort in Blyth’s study, which represented some of the earliest published clinical results for RA-UKA, dating back nearly 15 years. The temporal gap between these studies potentially reflects advancements in surgical techniques, implant design, and perioperative care, contributing to the observed improvement in functional outcomes. When compared to more recent studies, our RA-UKA results demonstrated comparable mean FJS results to the 6-month outcomes reported by Clement et al. [24]. However, our scores are 7 to 10 points lower than the 3- and 11-year results published by Catani’s group for reasons that are not immediately apparent to us [25,26,27,28].

Regarding RA-KA TKA, our FJS results align with those found in the existing literature, as reported by Abhari et al. [29].

To the best of our knowledge, only one pilot study has published results on postoperative activity levels or step counts following medial unicompartmental knee arthroplasty (mUKA) [30]. In that study, using traditional manual techniques, patients achieved their average daily step count at 6 weeks postoperatively, compared to 7 weeks in our investigation. This slight difference may be attributed to our implementation of a structured rehabilitation program delivered via a digital application with regular check-ins, enabling timely adjustments based on individual progress.

Our findings on step count recovery following total knee arthroplasty (TKA) align favorably with the existing literature. While previous studies have reported a return to preoperative step counts within 6 to 12 weeks post-surgery, our results indicate a slightly accelerated recovery, with patients regaining their preoperative step count at approximately 8 weeks postoperatively [31,32,33].

Besides this, there is an ongoing discussion on the clinical benefit of robotic-assisted knee arthroplasty. Especially for UKA, there are established data on decreased revision rates compared to manual instrumented surgeries, as well as on a faster rehabilitation [34,35]. Also, for TKA, there is growing evidence on the shorter length of hospital stay, less postoperative pain, and faster rehabilitation [36,37,38]. Unfortunately, many reviews or expert opinions look at 2-year outcome data with the established patient-reported outcome scores, and do not find a clinical benefit of newer techniques, such as robotic-assistance, individualized alignment, or partial knee arthroplasty, over the current standard of manual, mechanically aligned TKA. However, from both the patient’s perspective and a health-economical perspective, even a 2-week-faster recovery and an earlier return to work can have a positive effect and impact. Therefore, these types of data can contribute to the evaluation of new technology and treatment options, extending beyond the 2-year outcome data [39]. This was supported in our study with a new methodology like activity trackers.

This study, while insightful, is not without its inherent constraints. Several key limitations warrant careful consideration. The sample size is small, the follow-up period with activity trackers is short, and the data were analyzed retrospectively. In this context, the study was meant to be a pilot analysis on the potentially faster rehabilitation of UKA compared to TKA, and the methodology to observe regaining the preoperative activity with commercially available activity trackers. Second, as the participation in our activity tracker program was voluntary, there is only a very small group of patients included, and the data might be biased to more active and motivated patients. Plus, the individual activity differed a lot between the study participants (average daily preoperative step count ranging from 1374 to 17,933 steps), and the distribution of the morbidity state was different in the two groups. So, the data might not be transferable as a general finding for all UKA and TKA patients. The patient numbers were too low for a more specific subgroup-analysis. Third, as the activity trackers were given by the institution, this was not a completely unsupervised data acquisition. However, collecting data from patients’ own devices has more constraints in terms of data privacy as well as device comparability, and was therefore not used in our setting. Lastly, as there was no manually instrumented TKA control group, we were not able to analyze the specific effect of robotic arm-assisted surgery on the rehabilitation rate in this study.

While our focus on step count provides valuable insights into recovery trajectories, we acknowledge that rehabilitation is multifaceted and deserves equally multifaceted assessment approaches. We believe our findings represent an important contribution to understanding the differential recovery patterns between robotic arm-assisted mUKA and rKA-TKA procedures, while recognizing that future work would benefit from more comprehensive rehabilitation measurements.

## 5. Conclusions

Both robotic arm-assisted medial unicompartmental knee arthroplasty (mUKA) and robotic arm-assisted kinematically aligned total knee arthroplasty (rKA-TKA) patients achieved their average preoperative daily activity level within the first nine weeks after surgery. The mUKA patients, however, were likely to achieve this goal two weeks earlier, displaying a faster postoperative rehabilitation.

## Figures and Tables

**Table 1:** Descriptive analytics of demographic variables per group.

	rKA-TKA (n = 53)	mUKA (n = 359)	*p*-Value
Age (mean, SD)	68.6 (7.7)	66.7 (8.8)	0.283
Height (mean, SD)	169.1 (8.6)	171.5 (9.1)	0.227
Weight (mean, SD)	79.9 (17.6)	81.9 (14.4)	0.578
BMI (mean, SD)	27.8 (4.9)	27.7 (3.6)	0.912
Gender (F:M)	66%:34%	51%:49%	0.251
Side (L:R)	66%:34%	43%:57%	0.054
ASA (I:II:III)	13%:74%:13%	34%:46%:20%	0.023 *
Physical demand level (A:B:C:D)	19%:49%:26%:6%	9%:40%:42%:9%	0.274

* *p*-value < 0.05. BMI: body mass index; ASA: American Society of Anesthesiologists Status Classification System; Physical demand level: A, semisedentary; B, leisure activities; C, employed in light work; D, employed in strenuous work.

**Table 2:** Descriptive analytics of the step count per group and per interval. Group comparisons were performed using the Mann–Whitney U test.

		t0	t4	t5	t6	t7	t8	t9	t10
rKA-TKA	count	45	2	48	51	49	43	26	14
	median	3988	2911	1700	2370	2910	3383	4538	4814
	min	462	987	325	263	516	480	1241	2662
	max	13,297	4834	7608	11,307	11,317	11,523	12,822	14,755
	IQR	2674–6220	1949–3872	1158–2920	1067–4742	1433–5704	2133–7140	2982–6182	3314–5803
mUKA	count	29	3	27	30	33	27	14	5
	median	4315	2846	2217	3741	4730	4444	5479	5203
	min	2173	1971	667	1093	703	1230	1058	2082
	max	20,729	4254	7715	12,391	10,945	11,032	8948	14,336
	IQR	3540–6683	2409–3065	2619–5406	2619–5406	2556–5923	4044–7549	4308–7223	4328–8496
	*p*-value	0.128 ‡	N/A	0.277 ‡	0.015 ‡ *	0.048 ‡ *	0.193 ‡	0.275 ‡	N/A

t0: preoperative and t4–t10: postoperative week 4–week 10. ‡ Mann–Whitney U test (two-sided). * *p*-value < 0.05. N/A—significance tests not performed, as group sizes are too small.

**Table 3:** Descriptive analytics of PROMS at 2 months and 1 year per group.

	2 Months Follow-Up			1 Year Follow-Up		
	rKA-TKA	mUKA	*p*-Value	rKA-TKA	mUKA	*p*-Value
n =	49	35	-	49	34	-
KSS (mean, SD)	159.0 (24.7)	168.0 (21.5)	0.083 †	191.2 (14.0)	190.5 (12.3)	0.495 ‡
KOOS (mean, SD)	64.4 (15.7)	65.5 (13.3)	0.744 †	83.5 (12.9)	83.1 (10.9)	0.581 ‡
OKS (mean, SD)	34.7 (6.9)	34.5 (7.2)	0.881 †	43.4 (5.1)	42.5 (4.7)	0.214 ‡
EQ-5D-VAS (mean, SD)	82.8 (10.7)	80.0 (12.8)	0.42 ‡	88.9 (9.4)	86.2 (10.5)	0.253 ‡
EQ-5D-5L (mean, SD)	0.83 (0.19)	0.83 (0.12)	0.451 ‡	0.92 (0.12)	0.91 (0.13)	0.458 ‡
FJS (mean, SD)	50.0 (23.8)	48.1 (26.0)	0.734 †	76.1 (20.9)	75.4 (20.3)	0.791 ‡
FJS (median, IQR)	51.0 (34.4)	46.9 (37.0)	-	79.2 (28.1)	85.4 (29.2)	-

† Student’s *t*-test. ‡ Mann–Whitney U test (two-sided).

## Data Availability

Data are contained within the article.
